# Enteric Dysbiosis in Children With Autism Spectrum Disorder and Associated Response to Stress

**DOI:** 10.7759/cureus.53305

**Published:** 2024-01-31

**Authors:** Gesulla Cavanaugh, Jinbing Bai, Jaime L Tartar, Jue Lin, Tina Nunn, Naseer Sangwan, Diti Patel, Stachyse Stanis, Raina K Patel, Djellza Rrukiqi, Hannah Murphy

**Affiliations:** 1 Department of Nursing Research, Ron and Kathy Assaf College of Nursing, Nova Southeastern University, Davie, USA; 2 Nell Hodgson Woodruff School of Nursing, Emory University, Atlanta, USA; 3 Department of Psychology and Neuroscience, Nova Southeastern University, Davie, USA; 4 Department of Biochemistry and Biophysics, University of California San Francisco, San Francisco, USA; 5 Lerner Research Institute, Case Western Reserve University, Cleveland, USA; 6 Department of Allopathic Medicine, Nova Southeastern University Dr. Kiran C. Patel College of Allopathic Medicine, Fort Lauderdale, USA; 7 Department of Medicine, Dr. Kiran C. Patel College of Osteopathic Medicine, Nova Southeastern University, Davie, USA

**Keywords:** cognition, stress, gut-brain axis, gut microbiome, autism spectrum disorder

## Abstract

Background

Microbiome studies in humans, though limited, have facilitated the evaluation of the potential connection between the microbiome and brain function. Children with autism spectrum disorder (ASD) have several behavioral challenges and avoidant/restrictive food intake disorder, which may contribute to gut microbiome dysbiosis.

Aim

The aim of this study is to examine the extent to which the gut microbiome of children with ASD differs in comparison to children with neurotypical development (CWND) and to assess whether a probiotic intervention has the potential to influence the gut microbiome in mediating positive behavior change and stress regulation.

Methods

This pilot study collected data from three children with ASD and four CWND before and after a four-week probiotic intervention. Data collection included microbiome diversity screening from stool samples as well as the following biophysiological measures: salivary alpha-amylase (sAA) levels, response to simulated stressor and calming stimulus (behavior), including pulse rate, galvanic skin response, and pupil diameter (PD). In addition, telomere length was assessed. All measures, except for telomere length, were repeated after the four-week intervention on the ASD and CWND groups for pre-/post-comparison. Data analysis consisted of multivariate analyses, including ANOVA.

Results

While greater heterogeneity in the ASD group was evident in all measures, the gut microbiome of participants who received probiotic intervention differed from pretreatment results within and across the groups investigated. Further, the biophysiological parameter sAA displayed a significant increase between baseline and exposure to stress in both groups, whereas PD increased in both groups from baseline, *F*(11, 26615) = 123.43, *p* = 0.00.

Conclusion

Though gut microbiome diversity is diminished in children with ASD compared to CWND, the gap is narrowed following a brief probiotic intervention. The results suggest that probiotic interventions have the potential to rescue microbiome diversity and abundance, potentially supporting stress regulation in pediatric populations.

## Introduction

Between 2006 and 2020, the estimated prevalence of autism spectrum disorder (ASD) rose from one in 110 to one in 36 children [1]. ASD refers to a group of highly prevalent neurodevelopmental disorders characterized by persistent impairments in social interaction as well as the presence of restricted, repetitive patterns of behavior, activities, and interests, as defined by the Diagnostic and Statistical Manual of Mental Disorders, Fifth Edition (DSM-5) [2]. These impairments may lead to difficulties in social interactions and communication, impacting the ability to function in daily activities. While these characteristics are generally associated with ASD, this disorder has a heterogenous phenotype [3,4]. Individuals with ASD often have a wide range of symptoms and cognitive and physical functional levels; some may exhibit only a few observable traits, while others experience severe intellectual impairments requiring lifelong care. Although there is no single root cause of ASD, genetic and environmental factors, such as diet and stress, are involved in the pathogenesis of this condition [4,5].

Compared to children with neurotypical development (CWND), children with ASD are more likely to have comorbidities in addition to the core symptoms of autism [4,6]. Gastrointestinal (GI) disorders are among the more common medical comorbidities associated with ASD [6,7]. Common GI disturbances include diarrhea, constipation, chronic bloating, and gastroesophageal reflux. Further, studies have identified enteric dysbiosis in children with ASD as a characteristic of the disorder, suggesting that the gut-brain axis (GBA) may play a role in the onset of ASD [8,9].

Gut microbiome and gut-brain axis

There is growing recognition of the role of the enteric microbiome in modulating the central nervous system physiology. The central and enteric nervous systems communicate through bidirectional signaling pathways called the GBA. The strongest evidence for the role of microbiota in gut-brain signaling stems from a research study with germ-free mice, which showed fundamental neural processes, including myelination, neurogenesis, and microglia function, being dependent on the composition of the microbiome [10]. Overall, changes in the gut microbiome have been thought to affect the nervous system, with changes in the gut influencing early brain development [11].

Gut microbial signals to the brain

The composition of the gut microbiota can affect mood, communication, and cognition [10,12]. During dysbiosis, there is dysregulation of GBA pathways, leading to neuroinflammation and altered permeability of the blood-brain barrier [10]. Gut dysbiosis has been linked to numerous neuropsychiatric disorders [13,14]. Bastiaanssen et al. reported that patients with major depressive disorder have lower microbiome diversity compared to those without a neuropsychiatric disorder [15]. Clinically, it is thought that depressive episodes are correlated with a dysregulated hypothalamic-pituitary-adrenal (HPA) axis, and, conversely, mood stabilization is associated with an intact axis. Gareau et al. demonstrated that an administration of probiotics (i.e., *Lactobacillus* species) in rat pups could normalize corticosteroid release induced after maternal separation [16]. This effect is thought to be caused by altered enteric flora induced by the *Lactobacillus* species, which ultimately promote the release of serum corticosterone through modulation of the HPA axis. The products of the gut microbiota have also been suggested to play a role in the modulation of the GBA. For instance, the GBA helps regulate the release of the peptide hormone oxytocin, which is responsible for biological responses such as mood, stress, and pain [17]. Stress on the brain can alter microbiome diversity and abundance [18,19]. This effect is also observed in acute stress settings, independent of duration, causing major assaults on the gut microbiome [19]. Acute stressors increase the levels of proinflammatory cytokines interleukin-1β, interleukin-6, and interferon-γ, as well as decrease the mRNA expression of zonula occludens-2, which together serve to increase the permeability between colonic mucosal cells and damage the intestinal brush border [19,20].

Gut microbiome and autism spectrum disorder

While research has revealed variations in gut microbiome-derived metabolites and microbial taxonomy in children with ASD compared to CWND, there is no consensus on the precise microbial composition associated with ASD [20]. However, there is evidence of potential bidirectional relationships between the gut microbiome and social behavior [20]. Studies using germ-free mice and mouse models with ASD-like behaviors suggest that the gut microbiome influences social behavior [10,20]. When gut microbiota from individuals with ASD were transplanted into mice, these mice exhibited ASD-like behaviors, including increased repetitive actions and reduced sociability, indicating that altered gut microbiota can influence ASD-like behaviors in rodent models and individuals with ASD [20,21]. Of note, studies suggest that the absolute abundance of *Bifidobacterium* and *Blautia* species is significantly reduced in children with ASD compared to non-sibling controls [22,23], though this finding has not been consistent in the literature. It is also suggested that enteric dysbacteriosis may lead to the colonization of neurotoxin-producing bacteria in the intestines, contributing to ASD symptoms [4].

Despite recognizable patterns in the microbial composition, there is no predictive gut microbiome biomarker for ASD. Therefore, the purpose of this study was to examine whether microbiome diversity and abundance differ in children with ASD compared to CWND in ways that could be correlated to stress response, stress regulation, and behavior. Further, this study aimed to explore if microbiome diversity could be rescued with a short-term probiotic intervention and whether behavioral changes could be positively correlated with the restored microbiome.

## Materials and methods

Participants

Children with ASD and CWND were recruited to participate in a gut-microbiome intervention pilot after institutional review board approval. Data on seven children, three with ASD and four CWND, were analyzed to inform a larger study. The mean age was 5.5 ± 0.5 years for the ASD group (two males and one female) and 6.0 ± 0.5 years for the CWND group (three males and one female). ASD status was affirmed by the parents of participants who were between four and six years old. The parents of the participants were classified as follows: five as Caucasian (three children with ASD, two CWND) and two as mixed race (two CWND). To estimate percentage body fat, mean triceps skinfold measures (17.48 ± 1.0 mm, ASD; 18.73 ± 0.96 mm, CWND) were recorded for both groups. Verbal and written consent were obtained from the parents, while assent was obtained using a storyboard from the pediatric participants.

Data collection

Data were collected during two time points (pre-probiotic intervention and post-probiotic intervention). Eye-tracking and facial expression data were collected using the Tobii Pro Nano eye tracker (Tobii, Stockholm, Sweden) to evaluate the participants’ responses to a video scenario developed to elicit specific emotional responses. The video stimulus was five minutes long and was divided into four orderly frames: calibration, desensitization, mild stress-inducing, and relaxation. For the purpose of this pilot, the stress-inducing phase was composed of three short video clips (a low stressor with the sound of a child crying, a second low stressor with parents yelling, and a moderate stressor with children standing by ruins), and the relaxation phase was composed of five short video clips (a woman hugging a child, a male dancing with a baby in his arms, two Tom and Jerry cartoon clips, and a toddler laughing hysterically with a dog). Continuous pulse rate (PR) and galvanic skin response (GSR) were collected from all participants, though the duration of uninterrupted recordings was longer in the CWND group than in the ASD group. PR and perfusion index (Pi) were measured using the Masimo (RAD) pulse oximeter (Masimo Corporation, Irvine, CA) and were aggregated using Masimo TRACE software. GSR data were collected with the Tobii Pro Shimmer and synchronized with eye-tracking data within the Tobii Pro Lab software. During each participant’s initial visit, saliva was collected for telomere length (TL) as well as salivary alpha-amylase (sAA) analyses before exposure to the video stimulus and again five and 10 minutes after the stressor and five and 10 minutes after the relaxation stimulus. Parents assisted by collecting and freezing stool samples from their children prior to each visit, as well as by completing a survey to document food intake for the previous week. After the initial visit, pediatric participants followed a diet with the incorporation of healthy fruits and vegetables and a daily dosage regimen of three-fourths of a spoon of a powder pediatric probiotic blend (*Lactobacillus gasseri*, *Lactobacillus plantarum*, *Bifidobacterium lactis*, *Lactobacillus casei*, and *Lactobacillus acidophilus*) for four weeks, as tolerated. All pre- and post-data were compared between individuals and across ASD and CWND groups.

16S rRNA Gene Amplicon Sequencing and Bioinformatics

Stool samples were used for the process of 16S ribosomal RNA gene amplicon sequencing as previously published, followed by bioinformatics analyses [24-26]. The raw 16S amplicon sequences and associated metadata were demultiplexed using QIIME2 [27]. Next, individual FASTQ files, excluding non-biological nucleotides, underwent processing through the Divisive Amplicon Denoising Algorithm (DADA) pipeline [28]. The output of the DADA2 pipeline, represented by the feature table of amplicon sequence variants (ASVs), underwent subsequent analysis for alpha and beta diversity utilizing the phyloseq [29] and microbiomeSeq packages in R (R Foundation for Statistical Computing, Vienna, Austria). ANOVA was evaluated among sample categories with the assessment of α-diversity measures using the plot_anova_diversity function in the microbiomeSeq package. Permutational multivariate analysis of variance, involving 999 permutations, was performed on all principal coordinates obtained during canonical correspondence analysis (CCA) using the ordination function of the microbiomeSeq package. Additionally, pairwise correlation between microbiome genera and metabolomics data was executed using the microbiomeSeq package.

Salivary Alpha-Amylase

Saliva samples were run in duplicate and quantified using an sAA enzyme reaction protocol carried out according to the manufacturer’s instructions (Salimetrics LLC, Carlsbad, CA). A commercial microplate washer was used (BioTek 50, BioTek Instruments, Inc., Winooski, VT), and a BioTek ELx800 plate reader (BioTek Instruments, Inc., USA) was used to quantify the results. Final concentrations for each parameter were generated by Gen5 software (BioTek Instruments, Inc., USA), which collected absorbance at different reaction times.

Telomere Length

Genomic DNA was purified from saliva using the Agencourt DNAdvance Nucleic Acid Isolation kit (cat# A48705, Beckman Coulter, Inc., Brea, CA, USA). DNA was quantified by measuring OD260 with a NanoDrop 2000c Spectrophotometer (Nanodrop Products, Wilmington, DE, USA) and run on 0.8% agarose gels to confirm integrity. Samples that passed the quality control of OD260/OD280 between 1.7 and 2.0, concentration greater than 10 ng/mL, and no degradation were used for TL measurement.

Statistical analysis

Physiological and Eye-Tracking Data

Eye movement data harmonizing with GSR and PR data were analyzed using Tobii Pro Lab, Masimo TRACE, and JMP Pro 17; *p*-values <0.05 were considered significant for a 95% CI. The ratio of telomere signal versus single-copy gene signal from qPCR assay was obtained to yield average TL from the salivary samples. ANOVA models were generated to evaluate the differences between ASD and CWND based on pupil diameter (PD) change, GSR, PR, sAA levels, and TL measures.

Gut Microbiome Profile

Differential abundance analysis was conducted employing the random-forest algorithm as implemented in the DAtest package in R. In brief, differentially abundant methods were compared using metrics such as false discovery rate (FDR), the area under the (receiver operator) curve, empirical power (power), and false-positive rate. After benchmarking in accordance with DAtest standards, lefseq and ANOVA were identified as optimal methods for performing differential abundance analysis. Throughout the analysis, statistical significance was determined at *p* < 0.05, and *p*-values were adjusted for multiple comparisons using the Benjamini-Hochberg method to control the FDR [30]. To further characterize the associations, linear regression and Wilcoxon tests were employed on genera and ASV abundances against metadata variables, utilizing their base functions in R (version 4.1.2; R Core Team, 2021).

## Results

Biophysiological findings

**Figure 1:**
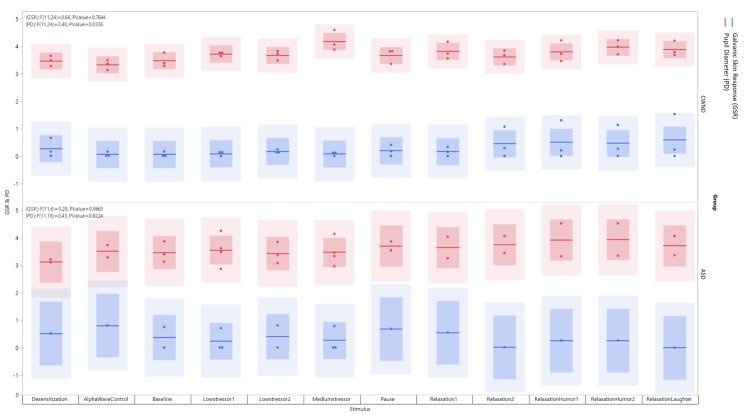
Bar graph showing aggregated GSR and PD measures over time during the presentation of stimulus for CWND and ASD groups. ASD, autism spectrum disorder; CWND, children with neurotypical development; GSR, galvanic skin response; PD, pupil diameter

A comprehensive analysis of physiological responses in study participants who received the stressful stimuli followed by relaxing stimuli revealed a heterogeneous response across multiple parameters. Statistical examination of GSR data from the Tobii Pro Lab analyzer suggested a nonsignificant increase in skin conductance from baseline when exposed to the stressor followed by the relaxation stimulus (*p* < 0.76). Though insignificant, this increase reflected heightened sympathetic nervous system activity in the CWND group in response to both stimuli. The findings were less consistent for the ASD group, in which the participants displayed no significant change in sympathetic responses under study conditions (Figure 1).

**Figure 2:**
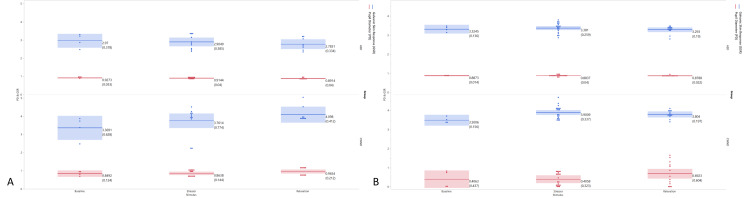
GSR and PD measures for combined stressor and relaxation stimuli. (A) Pre-intervention. (B) Post-intervention. ASD, autism spectrum disorder; CWND, children with neurotypical development; GSR, galvanic skin response; PD, pupil diameter

**Figure 3:**
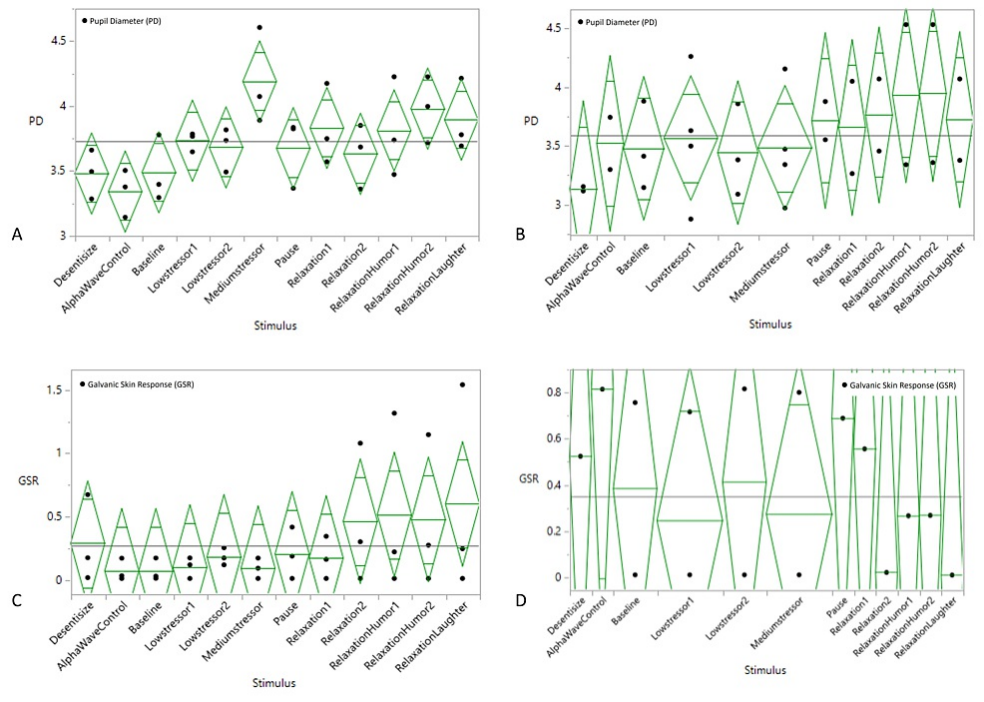
One-way analyses of variance for aggregated PD and GSR stratified by stimulus. (A) CWND (PD). (B) ASD (PD). (C) CWND (GSR). (D) ASD (GSR). ASD, autism spectrum disorder; CWND, children with neurotypical development; GSR, galvanic skin response; PD, pupil diameter

**Figure 4:**
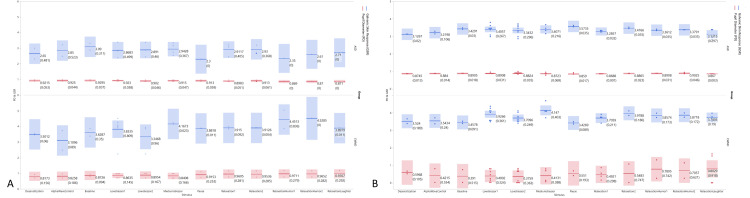
Mean GSR and PD for each stress and relaxation stimulus, including the break (pause) during which all devices continued to collect data on the participants. (A) Pre-intervention. (B) Post-intervention. ASD, autism spectrum disorder; CWND, children with neurotypical development; GSR, galvanic skin response; PD, pupil diameter

**Figure 5:**
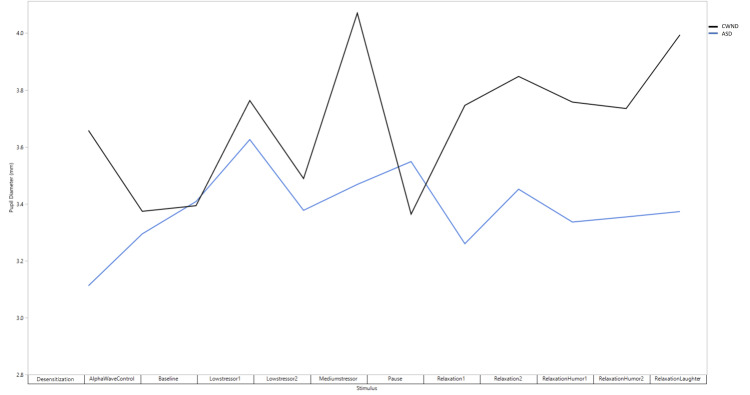
Pre-intervention comparison of ASD and CWND PD response to stimulus presentation, showing acute increase in PD (CWND) and acute decrease in PD (ASD) during exposure to moderate stressor. ASD, autism spectrum disorder; CWND, children with neurotypical development; GSR, galvanic skin response; PD, pupil diameter

Mean baseline PD pre-intervention was 3.40 ± 0.63 mm (CWND), 2.97 ± 0.38 mm (ASD), and 3.90 ± 0.15 mm (CWND), and post-intervention was 3.33 ± 0.14 mm (ASD). Unlike the findings of the skin conductance test, pre-intervention PD exhibited a statistically significant dilation from baseline in the CWND group (*p* < 0.04), indicating increased arousal and cognitive processing during both stressful and relaxing stimuli (3.76 ± 0.77 mm and 4.09 ± 0.41 mm, respectively), but not in the ASD group (2.91 ± 0.39 mm, 2.78 ± 0.33 mm) (Figure 2A), with similar results post-intervention (Figure 2B). One-way ANOVA analyses on all aggregated PD measures for the CWND group were statistically significant, *F*(11, 35) = 2.39, *p* = 0.04 (Figure 3A), but not for the ASD group, *F*(11, 29) = 0.43, *p* = 0.92 (Figure 3B), and not for GSR measures (Figures 3C, 3D). Significant pupil dilation from baseline was noted from both groups combined during exposure to the stimuli *F*(11, 26615) = 123.43, *p* = 0.00. Pre-intervention results indicated a greater increase from baseline in PD across stimuli in the CWND group, while results from the ASD group showed a variable decline in PD from baseline (Figure 4A). Post-intervention results for the CWND group also showed an increase in PD from baseline across the presented stimuli, while mostly similar PDs across multiple stimuli in the ASD group were detected (Figure 4B). There was, however, greater variability within the ASD group compared to the CWND group throughout progressive events during which participants from the CWND group exhibited predictable facial expression outcomes such as frowns, puzzled expressions (during the stressor), or pleasant expressions around the eyes, smiles, and laughter (during the relaxation phase). Collectively, participants with ASD did not exhibit these expected outcomes; however, further examination from the CWND and ASD groups pre-intervention revealed a peak in PD when the CWND were exposed to the moderate stressor, but the reverse was observed for the children with ASD (Figure 5). PD measures from both groups of children returned to baseline levels during the relaxation phase, then increased for the CWND during the relaxation with humor phase but were variable for the children with ASD.

 

**Figure 6:**
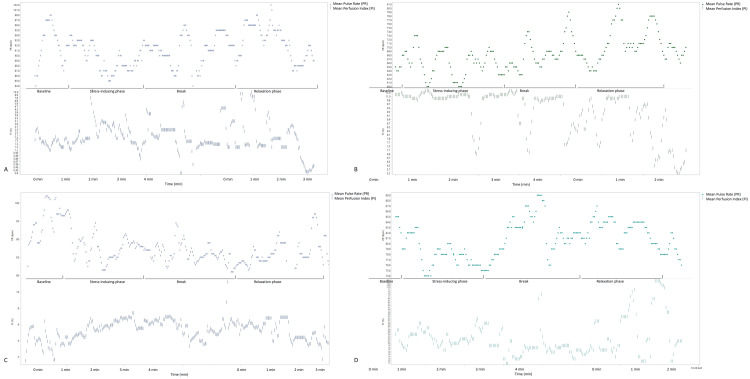
PR and Pi for CWND and ASD pre- and post-intervention. (A) CWND pre-intervention. (B) ASD pre-intervention. (C) CWND post-intervention. (D) ASD post-intervention. ASD, autism spectrum disorder; CWND, children with neurotypical development; Pi, perfusion index; PR, pulse rate

Furthermore, PR was elevated from baseline at the beginning of each stimulus, emphasizing a synchronized cardiovascular response to the stress and relaxation stimuli (Figure 6). PR also increased from baseline during the presentation of the relaxation with humor stimuli and during the break while saliva was collected from the participants. It was difficult to collect consistent pulse oximeter measures from the ASD group for an extended length of time due to behavioral challenges. Further, sAA values showed that, overall, the participants in both groups responded to the stressful stimulus. There was a significant difference in sAA baseline values (8.03 ± 0.17 U/mL in the CWND group and 16.08 ± 5.38 U/mL in the ASD group) and following presentation of the stress stimulus (10.77 ± 0.88 U/mL in the CWND group and 22.49 ± 2.12 U/mL in the ASD group), with p = 0.01 between the CWND and ASD groups. Both groups showed an overall increase in sAA in response to the stressor; sAA measures did not return to baseline levels post the relaxation phase for either group, either pre- or post-intervention, during the study timeframe. Regarding TL, five samples were processed, with four samples (two CWND and two ASD) yielding enough DNA for analysis. The average TL for the two ASD samples was lower compared to that of the two CWND samples.

Gut microbiome

**Figure 7:**
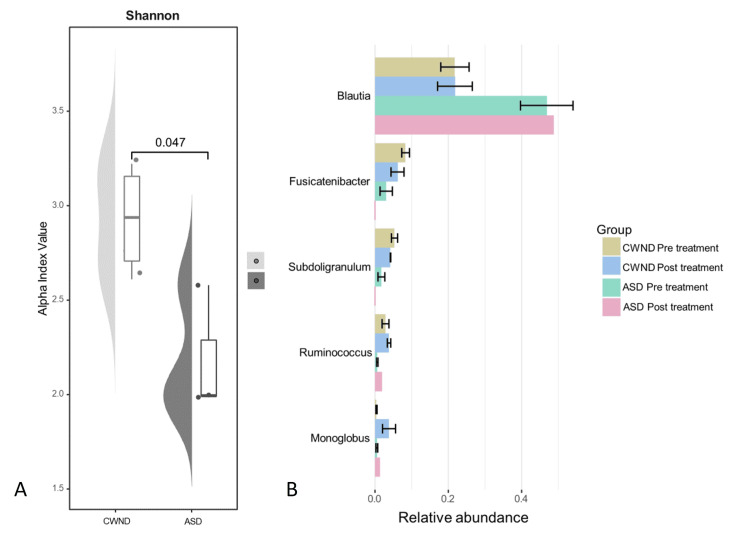
Microbiome profiling. (A) Alpha diversity of ASD and CWND before probiotic intervention. (B) Differential abundance analysis plot between ASD and CWND before and after probiotic intervention. ASD, autism spectrum disorder; CWND, children with neurotypical development

Analysis of the gut microbiome in the participants with ASD and CWND revealed significant differences between groups before and after the probiotic intervention. The Shannon diversity index for alpha diversity analysis suggests statistically significant differences in the gut microbiome diversity between the two groups (*p* < 0.05). CWND displayed greater microbial diversity compared to the participants with ASD (*p* < 0.05; Figure 7A). Consistent with these findings, the differential abundance analysis plot illustrating the percent abundance for distinct bacteria revealed that the gut microbiome of CWND was more balanced with beneficial bacteria than that of participants with ASD (Figure 7B).

**Figure 8:**
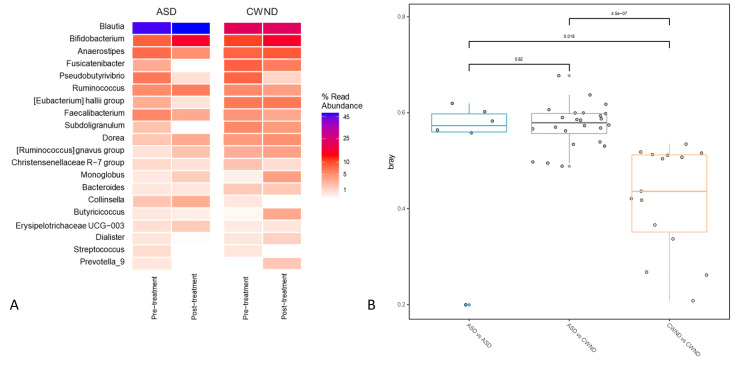
Microbial diversity and abundance measures. (A) Percent relative abundance; (B) Bray-Curtis microbial diversity for CWND and ASD groups before and after probiotic intervention. ASD, autism spectrum disorder; CWND, children with neurotypical development

**Figure 9:**
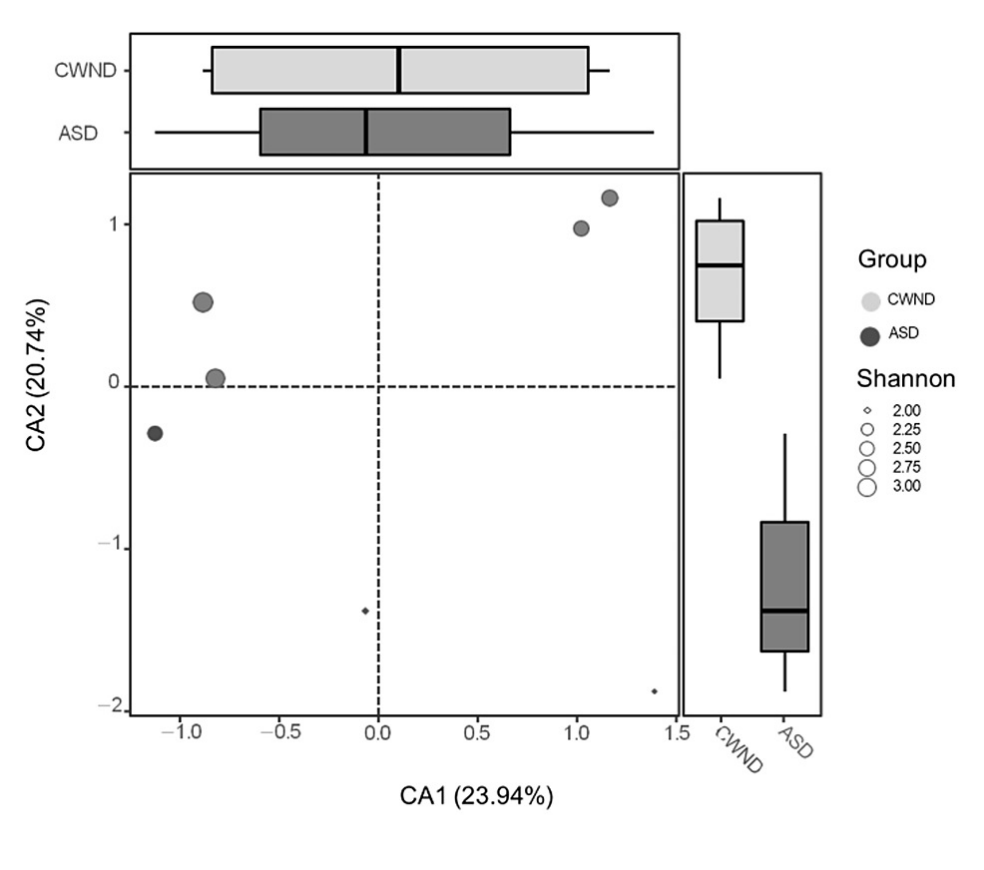
Canonical correspondence analysis plot demonstrating the microbiome profile from the ASD group compared to the CWND group. ASD, autism spectrum disorder; CA, correspondence analysis; CWND, children with neurotypical development

The Bray-Curtis similarity plot reflects higher microbiome abundance in the CWND group than in the ASD group pre- and post-probiotic intervention (Figure 8A), suggesting higher microbiome diversity in the CWND group. Pairwise dissimilarities between groups were higher for the CWND group and lower for the ASD group (Figure 8B). The CCA plot demonstrates a clear distinction between the gut microbiome profiles of participants with ASD and CWND (*p* < 0.05). This observed dissimilarity underscores a distinctive microbial community structure associated with ASD. The CCA captured the variations in microbial taxa contributing to the dissimilarities between groups, highlighting specific microbial signatures associated with this small group of pediatric participants with ASD (Figure 9).

## Discussion

A healthy gut microbiome plays an important role in metabolism, maintenance of the gut mucosal barrier, and immunomodulation [8]. Several factors shape the composition of the gut microbiome, including age, diet, antibiotics, and stressors such as infections [8,14]. Compelling clinical evidence supports the importance of a diverse and healthy gut microbial profile for optimum health, while assaults on the gut microbiome disrupt brain function and healthy development, increasing children’s susceptibility to various ailments. The findings from this pilot study are consistent with available clinical evidence, while conflicting results remain around *Bifidobacterium* and *Blautia* in children with ASD. The ASD group in this pilot study had a higher abundance of *Blautia* than the CWND group in contrast to findings from other studies [22,23]. Nonetheless, the results underscore the potential significance of gut microbiome dysregulation in the context of ASD, providing a foundation for further investigations into the mechanistic links between microbial composition and neurodevelopmental conditions.

The pilot study’s biophysiological results emphasize the integrated nature of physiological responses to stimuli that invoke either stress or pleasure, accentuating the coordination between autonomic nervous system activation, cognitive engagement, and cardiovascular reactivity. Further examination of participants from each studied group revealed that there are greater variations in children with ASD. More specifically, regarding PD, the ASD group experienced a significant decrease in PD during the presentation of the moderate stressor, while the CWND group displayed a significant increase in PD, which is an indicator of arousal due to stress or pleasure. The sharp reduction in PD from the ASD group may be an indicator of cognitive load as well as overload on the brain as it tries to receive and process stressful social stimuli. Following the four-week probiotic intervention, participants from both groups were less disturbed by the stressful stimuli based on facial expressions, behavior, and minor changes in PD, though both groups still had an acute response to the parents’ yelling stressor (low stressor) and children standing by ruins stressor (moderate stressor). Both groups enjoyed the cartoons pre- and post-intervention; however, CWND were more expressive with displaying their contentment.

The inherent challenges posed by the small sample and the associated limitations of this study are acknowledged, as they may amplify the impact of individual variations within each group. The feasibility of collecting and processing saliva for TL analysis yielded an 80% success rate, demonstrating the potential for assessing the biological health of the evaluated groups in future studies. However, because the results are limited due to the small sample size, it is not clear if any noted differences in the gut microbiome and in the participants’ responses to the presented stimuli after the probiotic intervention were due to a causal link or casual correlation, intrinsically a larger sample will yield more robust outcomes to inform practice.

## Conclusions

This study used a time series analysis of eye-tracking and microbiome data to compare children with ASD and CWND. The findings suggest that both groups experienced significant PD change diversely when exposed to moderate social stressors. Children with ASD exhibited a change in gut microbiome diversity and abundance after receiving a four-week probiotic and healthy diet intervention. Pre- and post-microbiome and biophysiological results further support the idea that a probiotic intervention may assist in replenishing the gut microbiome of children with ASD and may help attenuate acute physiological response to moderate stressors.

The results of this study emphasize the potential role of probiotics in restoring the gut microbiome and biophysiological responses even in children with ASD who are undernourished due to their AFRID. These results provide valuable insights into the intricate relationship between the gut microbiome and neurodevelopmental conditions, will inform a larger study, and can guide nutrition and behavioral therapies for pediatric patients.
